# Editorial: Highlights from conference of Biomaterials International 2023

**DOI:** 10.3389/fbioe.2025.1608462

**Published:** 2025-05-19

**Authors:** Shih-Jung Liu, Takao Hanawa, Alejandro Sosnik

**Affiliations:** ^1^ Department of Mechanical Engineering, Chang Gung University, Taoyuan, Taiwan; ^2^ Institute of Biomaterials and Bioengineering, Tokyo Medical and Dental University, Tokyo, Japan; ^3^ Department of Materials Science and Engineering, Technion Israel Institute of Technology, Haifa, Haifa, Israel

**Keywords:** biomaterials, tissue engineering, drug delivery, surface modification, biomechanics

## Introduction

The Biomaterials International 2023 Conference served as a pivotal forum for the global biomaterials community, bringing together researchers, clinicians, and industry experts to share groundbreaking advancements and foster collaborative efforts. Hosted in Sapporo, Japan, from July 30 to 3 August 2023, and organized by Chang Gung University and Tokyo Medical and Dental University, the conference underscored the interdisciplinary nature and rapid evolution of biomaterials science.

This special Research Topic, *“Highlights from Conference of Biomaterials International 2023,”* curates a selection of eight exemplary articles that encapsulate the innovative spirit and scientific rigor presented at the conference. These contributions span a diverse array of topics, reflecting the multifaceted landscape of biomaterials research.

## Decellularized tissue for soft-hard interface regeneration

Decellularized tissue, composed of extracellular matrix stripped of cellular components, offers a promising scaffold for tissue regeneration. Clinically, it has been used in various forms, including dermal and intestinal matrices, as prosthetic and covering materials. Suzuki et al. provides a review to explore how these tissues can support *in situ* regeneration by mimicking the structural and mechanical gradients of native tissue interfaces.

## Advancements in biomaterials for cancer therapeutics

One notable review explores the application of metal-organic frameworks (MOFs) in next-generation cancer treatments (Shano et al.). By adopting a biophysical perspective, the authors elucidate how MOFs can enhance drug delivery and efficacy, offering promising avenues for oncological interventions.

## pH-dependent control of carbonate apatite for artificial bone design

The success of artificial bone materials relies heavily on both their composition and internal architecture. In the study of Wang et al., carbonate apatite (CAp), a bone-like mineral, was synthesized from calcium carbonate under varying pH conditions to investigate how pH influences the resulting material. Advanced characterization techniques revealed that lower pH levels accelerated the transformation to CAp and altered its microstructure and degradation behavior.

## Innovations in implant surface modifications

The development of novel implant surfaces is critical for improving biocompatibility and integration. Research featured in this Research Topic investigates the use of phosphorylated pullulan for implant surface modification (Nagamoto et al.), demonstrating enhanced osteoconductivity and potential for improved clinical outcomes.

## Advances in biomedical alloys

The mechanical properties and biocompatibility of titanium-molybdenum (Ti-Mo) alloys are further enhanced through oxygen addition, as detailed in one of the original research articles (Kobayashi and Okano). This modification results in improved microstructure and mechanical performance, making Ti-Mo alloys more suitable for biomedical applications.

## Biomechanical insights into spinal surgery

A comparative biomechanical study examines the influence of various pilot hole profiles on pedicle screw fixation strength in both minimally invasive and traditional spinal surgeries (Li et al.). The findings provide valuable insights that could inform surgical techniques and improve patient outcomes.

## Deep learning for cranial implant design

The integration of deep learning techniques in biomedical engineering is exemplified by research on creating high-resolution 3D cranial implant geometries (Wu et al.). This approach streamlines the design process, offering personalized solutions for cranial reconstruction.

## Targeted drug delivery for lung cancer

An innovative study focuses on modified gefitinib-conjugated Fe_3_O_4_ nanoparticles for enhanced drug delivery in non-small cell lung cancer treatment (Thangudu et al.). The research provides an image-guided mechanistic analysis of tumor uptake, highlighting the potential for more effective and targeted therapies.

Collectively, these articles not only showcase the depth and breadth of research presented at Biomaterials International 2023 but also highlight the ongoing commitment of the biomaterials community to address complex healthcare challenges through innovative science and technology.

We extend our gratitude to all authors, reviewers, and conference organizers for their invaluable contributions to this Research Topic. It is our hope that this Research Topic serves as a catalyst for further discoveries and collaborations in the dynamic field of biomaterials.

For a comprehensive exploration of these studies, readers are encouraged to access the full articles available in this Research Topic.

